# Hydroxypyridinone Chelators: From Iron Scavenging to Radiopharmaceuticals for PET Imaging with Gallium-68

**DOI:** 10.3390/ijms18010116

**Published:** 2017-01-08

**Authors:** Ruslan Cusnir, Cinzia Imberti, Robert C. Hider, Philip J. Blower, Michelle T. Ma

**Affiliations:** 1 Division of Imaging Sciences and Biomedical Engineering, King’s College London, Fourth Floor Lambeth Wing, St. Thomas’ Hospital, London SE1 7EH, UK; ruslan.cusnir@kcl.ac.uk (R.C.); cinzia.imberti@kcl.ac.uk (C.I.); philip.blower@kcl.ac.uk (P.J.B.); 2 Institute of Pharmaceutical Science, King’s College London, Franklin-Wilkins Building, 150 Stamford Street, London SE1 9NH, UK; robert.hider@kcl.ac.uk

**Keywords:** hydroxypyridinone, deferiprone, gallium, iron overload, positron emission tomography, molecular imaging, bifunctional chelators

## Abstract

Derivatives of 3,4-hydroxypyridinones have been extensively studied for in vivo Fe^3+^ sequestration. Deferiprone, a 1,2-dimethyl-3,4-hydroxypyridinone, is now routinely used for clinical treatment of iron overload disease. Hexadentate tris(3,4-hydroxypyridinone) ligands (THP) complex Fe^3+^ at very low iron concentrations, and their high affinities for oxophilic trivalent metal ions have led to their development for new applications as bifunctional chelators for the positron emitting radiometal, ^68^Ga^3+^, which is clinically used for molecular imaging in positron emission tomography (PET). THP-peptide bioconjugates rapidly and quantitatively complex ^68^Ga^3+^ at ambient temperature, neutral pH and micromolar concentrations of ligand, making them amenable to kit-based radiosynthesis of ^68^Ga PET radiopharmaceuticals. ^68^Ga-labelled THP-peptides accumulate at target tissue in vivo, and are excreted largely via a renal pathway, providing high quality PET images.

## 1. Introduction

Positron emission tomography (PET) is a whole body diagnostic three-dimensional molecular imaging modality used in nuclear medicine that detects radiation arising from the decay of unstable positron-emitting radioisotopes. The availability of the positron-emitting isotope, gallium-68 (^68^Ga) from decay of ^68^Ge in a bench top ^68^Ge/^68^Ga generator is likely to have significant clinical impact on the use of PET for molecular imaging [1]. Clinical use of ^68^Ga receptor-targeting radiopharmaceuticals for neuroendocrine cancers (^68^Ga-DOTA-TATE) [2] and prostate cancers (^68^Ga-HBED-PSMA) [3] has changed patient management in centres that have routine access to such agents. ^68^Ga molecular imaging agents typically consist of chelators tethered to a receptor-targeted peptide, protein or small molecule. As Ga^3+^ can incorporate up to six ligands in an octahedral coordination sphere, hexadentate ligands are normally utilised. The Ga^3+^ ion has a high charge density, and is categorised as a “hard” Lewis acid, and so the majority of hexadentate ligands incorporate oxygen and/or nitrogen donor atoms. Both macrocyclic and acyclic chelators have been explored for ^68^Ga^3+^ complexation [1,4,5,6,7].

Clinical radiosyntheses of ^68^Ga-DOTA-TATE and ^68^Ga-HBED-PSMA involve heating at 80–100 °C for 5–20 min at pH 3–5 [1,4], followed by post-synthetic purification and work-up to remove impurities, unreacted ^68^Ga and reaction components that are not physiologically compatible. In the case of ^68^Ga-DOTA-TATE, heating is required for complexation of ^68^Ga^3+^. For ^68^Ga-HBED-PSMA, three geometric *cis*/*trans* isomers are possible, with each possible geometric isomer having a diastereomer. In this case, heating the reaction favours formation of the thermodynamically preferred species.

For ^68^Ga to be adopted in routine clinical practice, chelators that provide efficient and reproducible kit-based radiolabelling methods—preferably a single manipulation—are required. The chelators that we have developed based on hydroxypyridinones allow one-step quantitative ^68^Ga^3+^ radiolabelling at neutral pH and ambient temperature.

**Figure 1:**
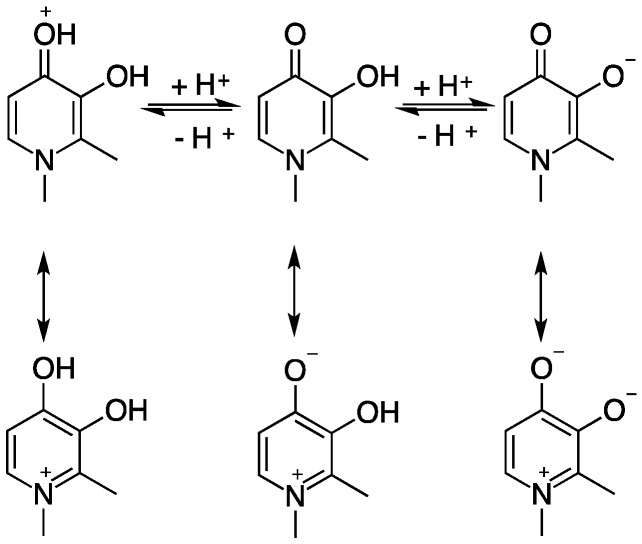
Deferiprone (**1**): Resonance structures at different protonation states [20].

Hydroxypyridinone (HP) ligands have high affinities for “hard” metal ions, and originally, the class of HPs described herein was developed extensively for therapeutic in vivo Fe^3+^ chelation in iron overload disease [8,9,10]. The bidentate HP ligand, deferiprone (**1**, Figure 1), is used routinely for this treatment [11]. Through chemical modification of substituents, Fe^3+^ affinity, hydrophilicity, metabolic stability and functionality of HPs can be tailored [10].

Ga^3+^ is an oxophilic metal ion, with a high charge density and ionic radius and coordination preferences similar to Fe^3+^. The (crystal) ionic radius for Ga^3+^ is 76 pm, and for high spin Fe^3+^ is 78.5 pm [12]. Recently, we have investigated the use of HPs for rapid and quantitative chelation of ^68^Ga^3+^ (as well as a long-lived PET isotope, ^89^Zr^4+^) and adapted a class of HPs to enable functionalisation with peptides and proteins for targeted molecular imaging [13,14,15,16].

Here we describe our research on the development of HPs for Fe^3+^ chelation, and how the design of such chelators is suitable for direct translation to ^68^Ga PET radiopharmaceuticals. It is not intended as an exhaustive review of chelators for either treatment of iron overload disease or complexation of radioisotopes of gallium used in nuclear medicine. The literature in both areas has been surveyed in depth in recent years in several excellent reviews and books [4,5,6,7,10,17,18,19].

## 2. Hydroxypyridinones

**Figure 2:**
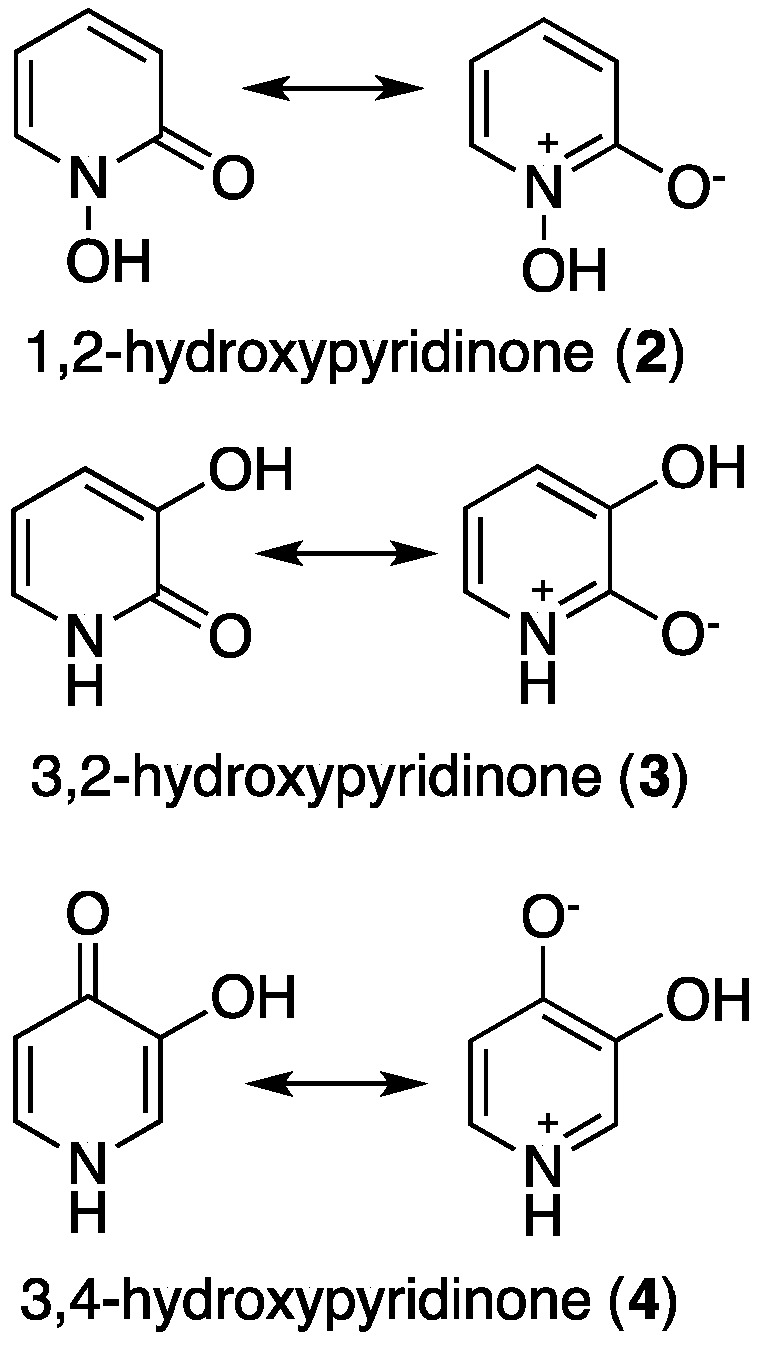
Resonance structures of 1,2-HP, 3,2-HP and 3,4-HP [21].

HPs consist of a six-membered aromatic *N*-heterocycle, with a hydroxyl and a ketone functionality. Varying relative positions of the hydroxyl and ketone functional groups within a bidentate HP molecule results in three types of hydroxypyridinones: 1,2-hydroxypyridinone (**2**); 3,2-hydroxypyridinone (**3**); and 3,4-hydroxypyridinone (**4**) (Figure 2). Neutral HPs can be protonated and deprotonated, with the first p*K*_a_ typically corresponding to protonation/deprotonation at the oxo group, and the second, at the hydroxyl group. Delocalisation of the electrons of the N^1^ ring atom leads to aromaticity (Figure 1 and Figure 2).

When HPs are deprotonated at the hydroxyl group, they are capable of complexing metal ions in a bidentate O_2_ mode, forming five-membered chelate rings. The relative positions of the hydroxyl and ketone groups, as well as ring substituents, influence p*K*_a_ values and metal binding affinities. In general, p*K*_a_ (the negative logarithm of the acid dissociation constant) and metal ion affinity values (both stepwise and cumulative stability constants of the metal-chelator complex) follow the order 3,4-HP > 3,2-HP and 1,2-HP (Table 1). This order reflects the relative decrease in delocalisation of the N^1^ atom lone pairs over the ring, and a corresponding decrease in charge density on the O atoms. The measurement of pM (negative logarithm of free metal concentration) is a more useful comparative measure for chelators than log *K*_a_ or affinity constants, as pM accounts for ligand basicity, denticity and protonation. Measurements are typically obtained at pH 7.4, with [total ligand] = 10^−5^ M and [total metal ion] = 10^−6^ M. Derivatives of high affinity 3,4-HPs have been extensively studied for iron-overload disease, and generally, 3,4-HPs have higher pM values than homologous 3,2- and 1,2-HPs. Additionally, 3,4-HPs are neutral at physiological pH, and when appropriately functionalised, can cross cell membranes.

**Figure 3:**
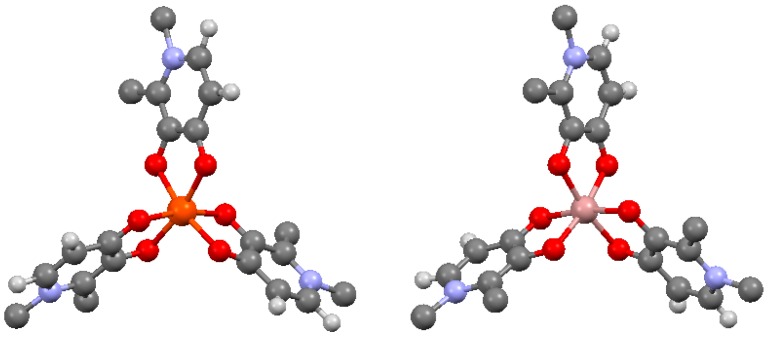
Representations of the molecular structures of [Fe(deferiprone)_3_] [31] (CCDC number 1183332) (**left**) and [Ga(deferiprone)_3_] (CCDC number 1164388) [29] (**right**). Only one stereoisomer is depicted for each complex. Orange—iron, pink—gallium, blue—ni trogen, grey—carbon, red—oxygen, and white—hydrogen. These diagrams (generated in Mercury software courtesy of the Cambridge Crystal Database) represent crystallographically determined structures. Hydrogen atoms of methyl groups are omitted for clarity.

3,4-HPs have high affinity for both Fe^3+^ and Ga^3+^, and form neutral 3:1 bidentate complexes with Fe^3+^ and Ga^3+^ (Figure 3, Table 2) (as well as Al^3+^, In^3+^, Cr^3+^, Mn^3+^ and Gd^3+^) [24,25,26,27,28,29,30,31]. Both the [Fe(deferiprone)_3_] [31] and [Ga(deferiprone)_3_] [29] complexes crystallise in the same octahedral geometries with comparable bond lengths and angles. In the three coordinating deferiprone ligands in both complexes, O^3^ atoms are *fac* to each other (see Table 3 for atom numbering scheme). Fe–O^3^ bond lengths are 1.998 Å, and Fe–O^4^ bond lengths, 2.038 Å while in the Ga analogue the corresponding Ga–O bond lengths are 1.967 Å, and 1.990 Å, respectively. In both complexes, both C–O bonds are intermediate between single and double bonds, with the C^4^–O^4^ bond shorter than the C^3^–O^3^ bond. Within each deferiprone ligand, bond angles are 80.91° for O^3^–Fe–O^4^, and 83.22° for O^3^–Ga–O^4^. Other metal ions (M = Al^3+^, In^3+^, Cr^3+^, Mn^3+^) form isostructural [M(deferiprone)_3_] complexes [27,28,29,30], and in all cases, *fac* geometries are observed in the solid state. However, ^1^H NMR studies on diamagnetic Ga^3+^ and Al^3+^ complexes suggest that conversion between *fac* and *mer* geometric isomers occurs in solution [30].

In living organisms, iron plays an essential role in transport of oxygen, electron transfer, and activation and functioning of enzymes. Iron transport and metabolism are tightly controlled in living organisms. In patients with iron overload disease, a disruption to iron homeostasis results in “free” iron, largely present as a citrate-albumin complex [32]. This iron pool gives rise to Fenton redox cycling between Fe^2+^ and Fe^3+^, producing free radicals that result in toxic tissue damage [33]:

Fe^2+^ + H_2_O_2_ → Fe^3+^ + HO· + OH^−^ (1)

Fe^3+^ + H_2_O_2_ → Fe^2+^ + HOO· + H^+^ (2)

Therapeutic chelators, including 3,4-HPs, are clinically used for sequestration of Fe^3+^ in vivo [7,8,9,10]. 3,4-HPs can be functionalised at the N^1^, C^2^, C^5^ or C^6^ positions of the ring (Table 3). An understanding of the effects of substitution is critical in optimizing 3,4-HP derivatives for either therapeutic Fe^3+^ chelation, or radiopharmaceutical Ga^3+^ chelation.

## 3. 3,4-Hydroxypyridinones for Fe^3+^ Complexation

### 3.1. Tailoring 3,4-HP Properties by Ring Substitution

Substitution at the C^2^ position results in pronounced effects on metal ion affinity. Alkylation increases pyridinone ring electron density, and increases p*K*_a_ values and log *K* and log *β*_3(Fe3+)_ constants (log *β*_3_ = 35.1 when R^2^ = H (**4**); log *β*_3_ = 37.2 when R^2^ = CH_3_ (**5**)) [20], although pFe^3+^ values are not markedly affected [26].

**Figure 4:**
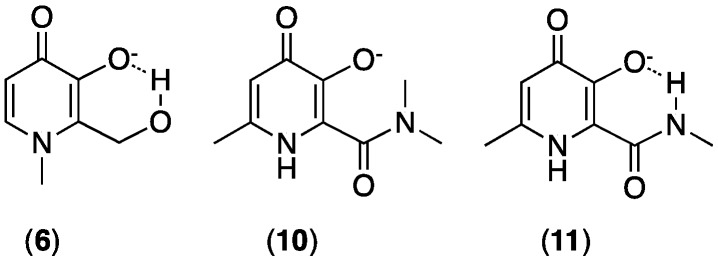
Intramolecular hydrogen bonding can stabilise ionised hydroxyl groups of 3,4-HPs.

Substitution of C^2^ alkyl groups for 1′-hydroxyalkyl groups decreases p*K*_a_ and log *β*_3(Fe3+)_ constants (compare **1** with **6**; and **7**, **8** and **9**) [34]. This decrease in affinity is a result of: (i) a decrease in ring electron density due to a negative induction effect of the C^2^ 1′-hydroxylalkyl group; and (ii) intramolecular hydrogen bonding between the C^2^ 1′-hydroxyalkyl group and the deprotonated C^3^ hydroxyl group (Figure 4). This hydrogen bonding stabilises the ionised C^3^ hydroxyl group. However, although Fe^3+^ affinity is decreased, the concurrent lowering of p*K*_a2_ actually results in an increase in pFe^3+^. The *decrease* in proton affinity favours the negative form that coordinates to Fe^3+^. In a side-by-side comparison at pH 7.4, pFe^3+^ values of the C^2^ 1′-hydroxyalkyl derivatives are higher than C^2^ alkyl derivatives [34].

Similar effects are also observed upon introduction of a C^2^ amido group (Figure 4) [38]. A combination of a negative induction effect and hydrogen bonding from the C^2^ amido NH to the C^3^ hydroxyl group decreases Fe^3+^ stability constants, but the concurrent decrease in p*K*_a_ serves to increase pFe^3+^ values at physiological pH relative to C^2^ alkyl derivatives. The C^2^ doubly alkylated N(CH_3_)_2_ amido group of **10** cannot form a hydrogen bond with the C^3^ hydroxyl group (Figure 4), and so its pFe^3+^ value is lower when compared to **11** bearing a singly alkylated NH(CH_3_) that can form a hydrogen bond (Table 3) [38].

In contrast to C^2^, varying alkyl substituents at the N^1^ position (H, methyl, ethyl) of 3,4-HP does not markedly affect the proton or metal ion affinity of 3,4-HPs (compounds **1**, **5**, **12** in Table 3) [20]. The exception to this is the C^2^ amido derivatives discussed above, where N^1^ alkylation sterically inhibits coplanarity of the C^2^ amido group with the HP ring, disrupting hydrogen bonding and thus decreasing pFe^3+^ values [38]. As such, N^1^ alkyl substitution can be a useful strategy for tailoring chelators’ lipophilicity, cell permeability [20], in vivo biodistribution [34], and rates of metabolism [39] without deleterious effects on metal ion affinity. Substitution at N^1^ sites has also been utilised to functionalise 3,4-HPs with fluorescent tags [40] or biological vectors [41].

Alkyl substitution at the C^5^ position increases 3,4-HP affinity for Fe^3+^, however as there is a concurrent increase in p*K*_a2_, pFe^3+^ does not increase relative to derivatives that do not contain a C^5^ alkyl group (compounds **13** and **14**) [36]. There are no studies directly comparing and quantifying the effect of C^6^ substitution, but existing data of C^6^ methylated derivatives suggest that alkylation has little influence on 3,4-HP metal affinity [36].

**Figure 5:**
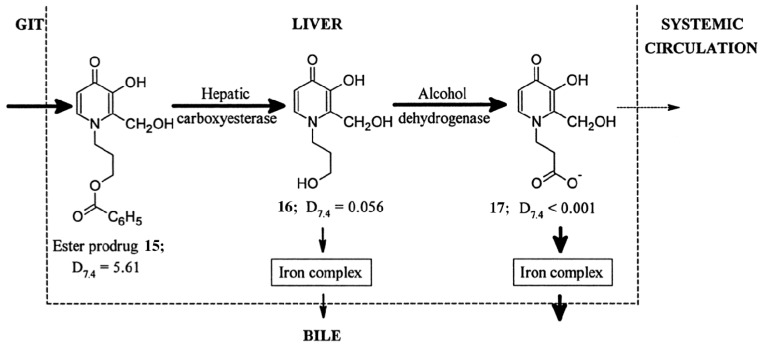
Schematic representation of the use of hydrophobic esters to enhance both the absorption from the gastrointestinal tract (GIT) and the hepatic extraction of the chelator. Subsequent intracellular hydrolysis occurs yielding a hydrophilic chelator which can undergo further metabolism to the extremely hydrophilic negatively charged 1-carboxyalkyl metabolite in the liver. Reprinted with permission from Liu, Z.D.; et al. Synthesis, physicochemical characterisation, and biological evaluation of 2-(1′-hydroxyalkyl)-3-hydroxypyridin-4-ones: Novel iron chelators with enhanced pFe^3+^ values. *J. Med. Chem.* **1999**, *42*, 4814–4823. Copyright American Chemical Society.

Increasing the lipophilicity of 3,4-HPs increases their cell membrane permeability. Higher intracellular 3,4-HP accumulation results in greater intracellular Fe^3+^ scavenging, however excessive cellular uptake of 3,4-HPs results in toxicity [20]. A prodrug strategy that involves oral administration of an N^1^-substituted hydrophobic ester 3,4-HP (**15**, Figure 5) has proved particularly successful in scavenging iron in a preclinical iron overloaded rat model [34]. In iron overload disease, excess iron is stored in the liver, and the rat model mimics this. The ester compound **15** is absorbed effectively in the gastrointestinal tract, and delivered to the liver, where it enters target iron-overloaded hepatocyte cells. The compound is proposed to undergo ester hydrolysis and metabolism intracellularly (**16**, **17**) prior to complexing Fe^3+^ [20]. The complex is then excreted, recovering excess iron from diseased animals efficiently. In contrast, when the more hydrophilic N^1^ propyl alcohol derivative **16** (Figure 5) is directly administered to rats, absorption is less effective, and Fe^3+^ recovery is lower.

### 3.2. Deferiprone, Desferrioxamine and Deferasirox

Deferiprone (or Ferriprox) (**1**), developed by Hider and colleagues, has been clinically approved (in Europe in 1999, and the USA, in 2011) for treatment of iron overload diseases, including hemochromatosis and transfusion-dependant thalassemia [42]. The advantage of deferiprone is that it is both orally active and effective at sequestering Fe^3+^ from the blood stream, heart (where iron overload toxicity can be fatal) and liver (where excess iron is stored).

**Figure 6:**
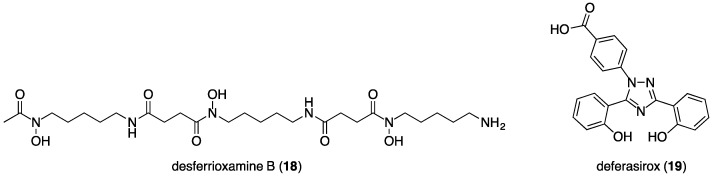
Structure of desferrioxamine B (DFO) and deferasirox.

Two other approved treatments for iron overload are available. The first is the hexadentate hydroxamate chelator, desferrioxamine-B (DFO, **18**, Figure 6), which forms a complex with Fe^3+^ with a metal to ligand stoichiometry of 1:1 and an overall charge of +1 under physiological conditions. DFO requires parenteral infusion over 8–12 h, several times a week, as it is not orally absorbed [42]. Clinical studies have demonstrated that either deferiprone alone, or a combination of deferiprone and DFO, are more effective therapies for myocardial iron overload than DFO alone. Additionally, a combination of oral deferiprone and parenteral DFO therapies is more viable for patients than parenteral DFO therapy alone, as combination therapy results in fewer parenteral infusions.

Like deferiprone, deferasirox [43] (**19**, Figure 6) is an effective orally active treatment for iron overload with clinical approval (in Europe in 2006 and the USA in 2005). It is a tridentate chelator, with Fe^3+^ complexes bearing a 3-charge at physiological pH. Data from clinical comparisons of the efficacy of deferiprone and deferasirox are conflicting and inconclusive, possibly due to variations in doses of the two treatments [44,45,46,47]. Some clinical data suggest that deferiprone is more effective at reducing iron levels in cardiac tissue [46,47].

### 3.3. Synthesis of 3,4-HPs

The structural diversity of 3,4-HPs is a result of extensive synthetic research that spans several decades. It is beyond the scope of this review to describe all of these synthetic routes in great detail, but it is worth highlighting common starting routes, and routes that give rise to N^1^- and C^2^-substituted 3,4-HPs that are important precursors for new, bioactive compounds.

**Scheme 1:**
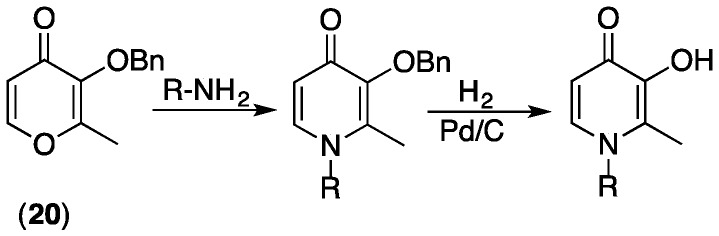
Synthetic route for N^1^-substituted 3,4-HPs.

Pyranones such as maltol (**20**) containing a benzyl (Bn) protecting group can simply be converted to pyridinones by reaction with primary amines (Scheme 1) [20]. This allows diverse substitution at N^1^, including incorporation of reactive groups such as carboxylates or primary amines that lead to further functionalization [20,40].

**Scheme 2:**
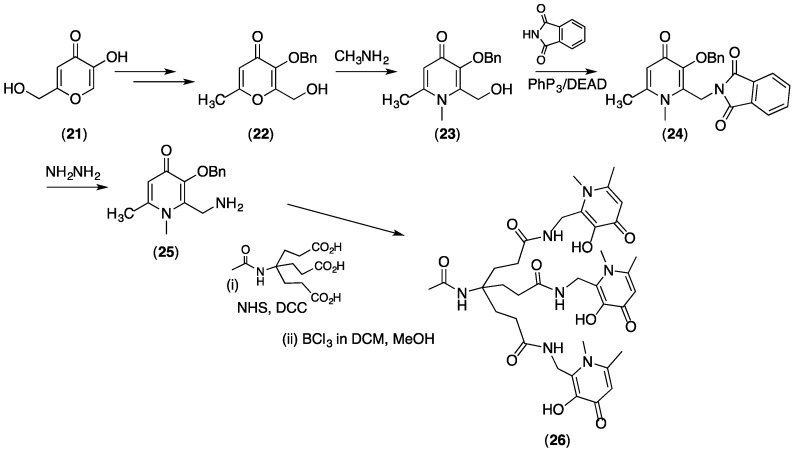
Synthesis of a useful 3,4-HP with a reactive amine (**25**) that is used for synthesis of THP-Ac (**26**).

Compound **22** is a pyranone containing a Bn protecting group, and a reactive alcohol. It is a key synthetic precursor enabling versatile manipulation of functionality at the C^2^ position of 3,4-HPs (Scheme 2). It is synthesised in high yields from the commercially available and inexpensive precursor, kojic acid (**21**) in four steps [48,49]. As discussed above, the Bn-protected **22** can be converted to Bn-protected pyridinone **23** by reaction with methylamine [49]. Protection of the ethyl alcohol group (and subsequent deprotection) increases yields in this reaction. A Mitsunobu reaction of **23** with phthalamide gives a Bn-protected pyridinone (**24**) that contains a phthalamide at the C^2^ position, that can be simply converted to a primary amine (**25**) [49]. Bn-protected **25** is a very useful precursor for preparation of tris(hydroxypyridinones) such as **26** (see below).

**Scheme 3:**
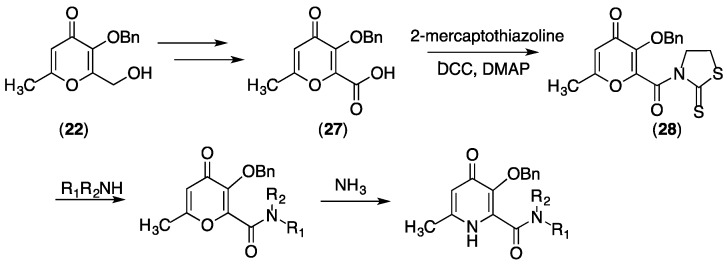
Synthesis of 3,4-HP precursors containing primary and secondary amides.

Alternatively, **22** can be converted at the alcohol to a carboxylic acid in two steps to give pyranone **27** (Scheme 3) [48]. Compound **27** can then be coupled with 2-mercaptothiazoline using appropriate reagents to give pyranone **28** that contains an active amide. Compound **28** can be reacted with primary or secondary amines, resulting in pyranone amide derivatives that can be converted to Bn-protected pyridinones by reaction with methylamine or ammonia [38,48]. For example, Bn-protected precursors to compounds **10** and **11** are prepared in this fashion.

**Scheme 4:**
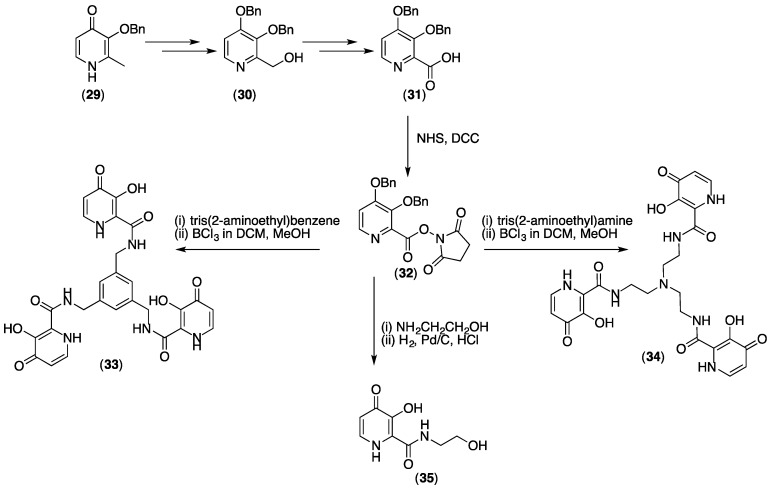
Synthesis of a 3,4-HP containing a reactive carboxylate that allows synthesis of THPs **33** and **34**.

Reactive carboxylates are synthetically accessible from Bn-protected pyridinones (Scheme 4) [50]. Bn-protected **29** can be further protected, and the methyl group substituted to ultimately yield **30**. Compound **30** can be converted to a carboxylate, yielding **31**. The carboxylate group of **31** can be further activated with an *N*-hydroxysuccinimide if required (**32**). Such derivatives have been used to prepare tris(hydroxypyridinones) such as **33** and **34** as well as other amide derivatives (**35**) [50].

Deprotection of Bn-protected hydroxyl groups proceeds via either hydrogenation reactions (catalysed by palladium on carbon) followed by acidification, or treatment with dissolved boron trichloride in an aprotic solvent, followed by addition of an alcohol (Scheme 1, Scheme 2 and Scheme 4).

## 4. Hexadentate Tris(hydroxypyridinone) Ligands

### 4.1. Topology and Fe^3+^ Affinity

Incorporation of three bidentate 3,4-HP ligands into a tripodal construct provides hexadentate tris(hydroxypyridinone) ligands (THPs) that, in a suitably designed scaffold, saturate the coordination sphere of octahedral metal ions. THPs have been designed for applications in gastrointestinal scavenging of Fe^3+^ [51], antimicrobial activity (via deprivation of microbes’ Fe^3+^ pool) [52,53], and fluorescence imaging of cellular Fe^3+^ distribution [54]. The topology of tripodal THPs is critically important to formation of octahedral complexes with 1:1 stoichiometry. The backbone of the tripod should be connected *ortho* to a coordinating O atom [55].

The synthesis of the first generation of THP ligands utilised 3,4-HP groups with a C^2^ carboxylate substitution (**32**), allowing reaction with tripodal polyamines to yield compounds such as **33** and **34** (Scheme 4). In **33** and **34**, 3,4-HP units are attached via a C^2^ amide group, and fulfil the topology requirements outlined above for formation of hexadentate compounds with 1:1 stoichiometry. For compound **34**, log *K*_1_ = 30.7, whereas for the bidentate homologue **35**, log *β*_3_ = 31.4 [50]. In this case, the affinity of the bidentate 3,4-HP for Fe^3+^ is already optimal, and incorporation into a hexadentate form does not result in an increase in thermodynamic stability, as might normally be expected for an increase in ligand denticity and accompanying lower entropic costs. The pFe^3+^ of **34** (30.5 at pH 7.4) is higher than the pFe^3+^ of **35** (22.0) [50]. This arises because the formation constant of a hexadentate complex of **34** has only a first order dependence on ligand concentration (1:1 ligand to metal stoichiometry), whereas that of **35** necessarily has third order dependence on free ligand concentration (3:1 ligand to metal stoichiometry). In solutions of **34** where the hexadentate ligand concentration = 10 µM, the total concentration of single 3,4-HP units is three times greater than in solutions of **35** where the bidentate ligand concentration = 10 µM.

Instead of polyamine tripods, the next generation of THP ligands derivatised tripodal carboxylate groups, using 3,4-HP units with a C^2^ aminomethyl substituent (**25**, Scheme 2) [51]. In the resulting compounds, for example THP-Ac (**26**, Scheme 2), each 3,4-HP group is tethered to the tripod via an amidomethyl linker at the C^2^ position. The N^1^ and C^6^ positions are methylated. With the exception of C^6^-methylation, THP-Ac’s single 3,4-HP unit is structurally similar to deferiprone. For THP-Ac, log *K*_1_ = 32.52 and pFe^3+^ = 28.47 [51].

The incorporation of three 3,4-HP groups into a tripodal ligand also has implications for the *lability* of hexadentate complexes. For example, in [Fe(THP-Ac)], dissociation of a single 3,4-HP unit is likely to be followed by its rapid recoordination to the metal centre. During dissociation, this 3,4-HP unit will remain *spatially close* to the metal centre, as the other two 3,4-HP groups, to which it is covalently tethered, are likely to still be bound to Fe^3+^. On the other hand, in [Fe(deferiprone)_3_], if a deferiprone ligand dissociates, it has a lower probability of recoordinating to the same metal centre, as it is not anchored to any other coordinating ligands. The activation energy barrier to dissociation of [Fe(THP-Ac)] is higher than that of [Fe(deferiprone)_3_], and [Fe(THP-Ac)] is more kinetically inert than [Fe(deferiprone)_3_].

### 4.2. Dendrimers Based on THP Units

**Figure 7:**
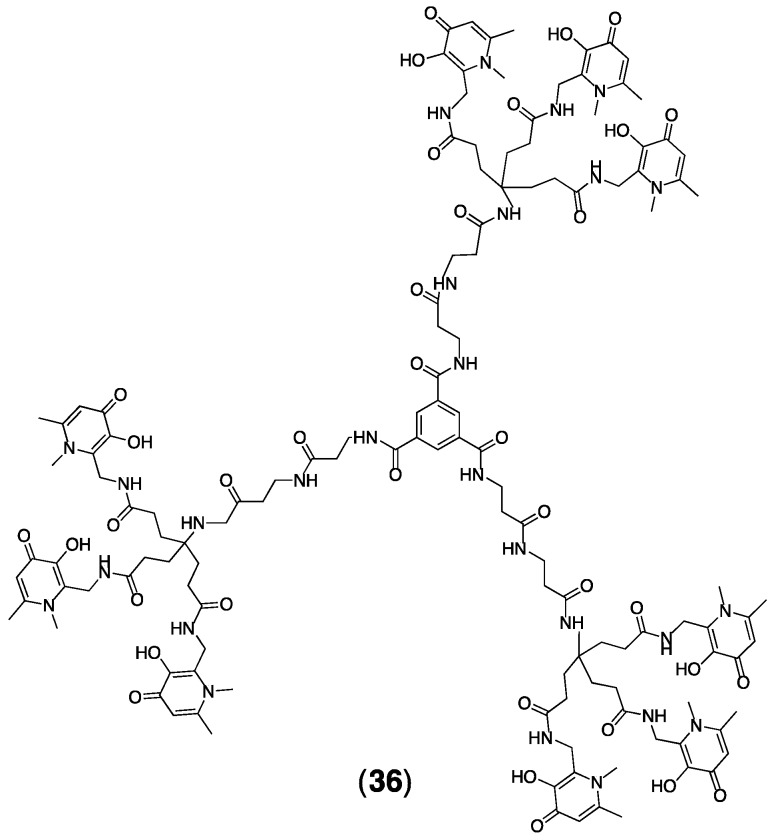
Dendritic 3,4-HPs such as (**36**) can coordinate multiple Fe^3+^ ions.

Dendritic THP molecules, for example **36**, have incorporated between three and six THP groups, allowing coordination of between three and six equivalents of coordinatively saturated Fe^3+^ per molecule [51,56]. Dendrimers have been prepared from both **25** and **32**. Affinity constants *as well as* pFe^3+^ values of dendrimer **36** (Figure 7) (log *K*_1_ = 32.74, pFe^3+^ = 28.69) and other derivatives do not meaningfully deviate from values for the single THP-Ac homologue **26**, indicating that maximum Fe^3+^ binding efficiency is achieved using 3,4-HP motifs in a tripodal THP topology.

### 4.3. Derivatising THP Ligands

**Figure 8:**
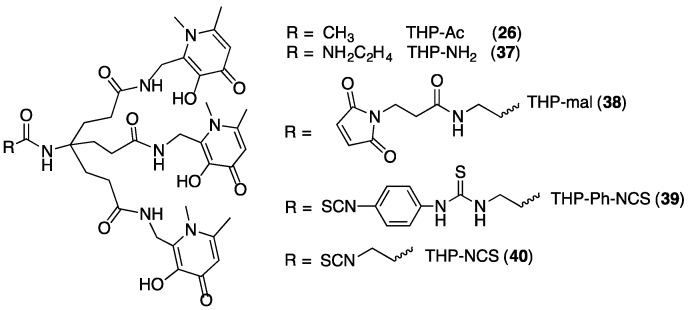
THP derivatives.

THP compounds of the same topology and substitution as THP-Ac (**26**) are synthetically accessible from the β-alanine derivative, THP-NH_2_ (**37**, Figure 8) using similar reaction routes to that described in Scheme 2 [51]. THP derivatives such as **33** or **34** are less amenable to derivatisation in this fashion. The presence of an apical primary amine in THP-NH_2_ allows attachment to dendritic scaffolds [51], biomolecules [13,14,15,16], polymer units [57], and fluorophores [54]. This ability to functionalise THP compounds makes them attractive for technological applications where trivalent metal ion complexes of high affinity and kinetic stability are required. We have explored THP derivatives for PET imaging with Ga^3+^.

## 5. Tris(hydroxypyridinone) Ligands for Radiolabelling with ^68^Ga^3+^

### 5.1. Radiolabelling Peptides with ^68^Ga for PET Imaging: The Case for Tris(hydroxypyridinone) Derivatives

Peptide-based molecular imaging agents can rapidly accumulate at target tissue and clear from circulation within 1–2 h. The 68 min half-life and positron emission properties of ^68^Ga (β^+^ 90%, *E*_max_ = 1880 KeV) match these requirements for PET imaging of peptide receptor expression. Moreover, ^68^Ga is conveniently available by elution of the ^68^Ge/^68^Ga generator to produce no-carrier-added solutions of ^68^Ga^3+^ in hydrochloric acid [58].

Hydrated Ga^3+^ species such as [Ga(H_2_O)_6_]^3+^ exist in aqueous solution below pH 4. As the pH is raised above 4, the poorly soluble hydroxide species Ga(OH)_3_ predominates in solution, until pH > 6.3, where tetradentate [Ga(OH)_4_]^−^ predominates [59,60]. For efficient ^68^Ga^3+^ radiolabelling of chelate-peptide conjugates at neutral or near neutral pH, chelate complex formation must effectively compete with unreactive ^68^Ga-colloid formation. Preferably, the rate of chelation will be diffusion-controlled, so that complex formation outcompetes ^68^Ga^3+^ colloid formation. Additionally, the amounts of ^68^Ga eluted from clinical generators are in the range of 200–2000 MBq, approximately equivalent to 2–20 pmol of ^68^Ga^3+^ in 1–5 mL of solution. Highly efficient chelators are required to quantitatively complex such low concentrations of metal ion without using excessively high chelator concentration. In clinical formulations of chelator bioconjugates, low concentrations of chelator-peptide conjugate are important. Clinical formulations do not usually separate unlabelled bioconjugate from labelled conjugate, and large amounts of unlabelled conjugate can lead to saturation of target receptors in vivo.

As THP ligands have extraordinarily high pFe^3+^ values, and Ga^3+^ has similar coordination preferences to Fe^3+^, it was reasoned that such chelators could be very efficient at quantitatively coordinating ^68^Ga^3+^ at low chelator concentrations [13]. The acyclic nature of THP, and hence flexibility compared to macrocyclic ligands, results in low activation barriers to complexation, resulting in rapid rates of reaction at room temperature.

It is also critical that the ^68^Ga^3+^ complex is sufficiently kinetically stable over the period of time required for imaging (1–2 h) to withstand transchelation by competing endogenous proteins, such as transferrin, and other ligands that compete for Ga^3+^ in vivo [4]. The iron transport protein transferrin is abundant in serum and its two metal binding sites have high affinity for Fe^3+^ (log *β*_1_ = 22.8, log *β*_2_ = 44.3) and Ga^3+^ (log *β*_1_ = 20.3, log *β*_2_ = 39.6) [61]. Transchelation of ^68^Ga^3+^ to endogenous ligands results in increased non-target tissue uptake and lower tumour/diseased tissue uptake, decreasing PET image quality.

As 3,4-HP and THP derivatives can bind Fe^3+^ under physiological conditions, with the resulting complexes excreted, it was hypothesised that a THP Ga^3+^ complex could be sufficiently stable in vivo. Mouse biodistribution studies with the γ-emitting isotope, ^67^Ga^3+^ (half-life = 78 h), comparing the biodistribution of [^67^Ga(deferiprone)_3_] with [^67^Ga(citrate)_3_] show that mice administered the deferiprone complex intravenously have lower ^67^Ga blood activity one day post-administration than animals administered the citrate complex [37]. Subsequent studies demonstrate that ^67^Ga: (i) clears more rapidly from animals administered the deferiprone complex compared to animals administered the citrate complex; and (ii) clears predominantly via a renal pathway in animals administered the deferiprone complex [37,62]. N^1^-functionalised derivatives of 3,4-HP are also efficacious at sequestering ^67^Ga^3+^ in vivo, with biodistribution of radioactivity modified by N^1^ substituents [62]. Such results suggest that [^67^Ga(deferiprone)_3_] has appreciable stability in vivo, and that 3,4-HPs can effectively compete with endogenous protein ligands for Ga^3+^ [37,62].

### 5.2. Tris(hydroxypyridinone) Bioconjugates

**Figure 9:**
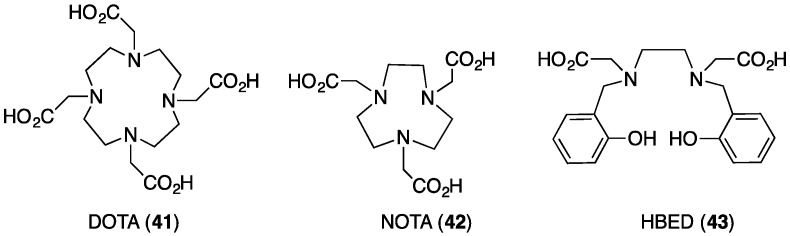
Chelators for ^68^Ga^3+^.

**Figure 10:**
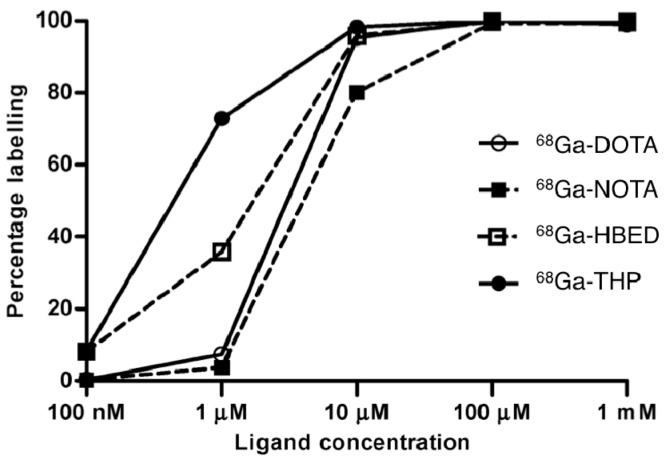
Radiolabelling yield versus ligand concentration for ^68^Ga-DOTA (pH 4.4, 30 min, 100 °C), ^68^Ga-NOTA (pH 3.6, 10 min, room temperature), ^68^Ga-HBED (pH 4.6, 10 min, room temperature) and ^68^Ga-THP, (pH 6.5, 5 min, room temperature). All experiments were conducted with the same batch of ^68^Ga eluate. All radiolabelling buffers were 0.2 M acetic acid/sodium acetate. Reprinted with permission from Berry, D.J.; et al. Efficient bifunctional gallium-68 chelators for positron emission tomography: tris(hydroxypyridinone) ligands. *Chem. Commun.* **2011**, *47*, 7068–7070. Copyright Royal Society of Chemistry.

We first reported the utility of THP-Ac (**26**, Figure 8) as a basis for highly efficient ^68^Ga labelling under very mild conditions after undertaking side-by-side comparisons of THP-Ac with chelators already commonly used to complex ^68^Ga^3+^: macrocyclic derivatives DOTA (**41**) (1,4,7,10-tetraazacyclododecane-1,4,7,10-tetraacetic acid) and NOTA (**42**) (1,4,7-triazacyclononane-1,4,7-triacetic acid), and the acyclic chelator HBED (**43**) (bis(2-hydroxybenzyl)ethylenediaminediacetic acid) (Figure 9) [13]. Each chelator was reacted with generator-produced ^68^Ga^3+^ at progressively lower chelator concentrations (100 nM–1 mM, each 100 µL corresponding to amounts of 10 pmol–100 nmol) (Figure 10), with the reasoning that the most efficient, rapidly complexing chelators would maintain high labelling efficiency at the lowest ligand concentrations. Optimised reaction conditions (as reported in the radiochemical literature) for each chelator were employed. At a concentration of 10 µM, THP-Ac complexes ^68^Ga^3+^ in 5 min in >98% radiochemical yield at pH 6.5 at room temperature. Radiochemical yields for DOTA are >95% at the same chelator concentration, but this requires heating at 100 °C at pH 4.4. NOTA has been reported to coordinate ^68^Ga^3+^ efficiently at room temperature, but at 10 µM concentration at room temperature, pH 4.4, radiochemical yields only average 80%. At 10 µM, HBED is able to complex ^68^Ga^3+^ in >96% radiochemical yield at pH 4.6. Whilst HBED radiochemical yields under acidic conditions are comparable to those of THP-Ac at near neutral pH, HBED forms geometric isomers when complexed to Ga^3+^ [4]. From a regulatory perspective, the presence of different geometric isomers is undesirable, as it is possible that the different isomers have different pharmacological profiles.

The β-alanine-derived compound, THP-NH_2_ (**37**) [51], can be functionalised for bioconjugation via a maleimide [13,16] and isothiocyanates [14,15] (Figure 8). The bifunctional chelators THP-mal (**38**), THP-Ph-NCS (**39**) and THP-NCS (**40**) have all been conjugated to biomolecules. Amide conjugation of THP-NH_2_ (**37**) to activated carboxylates of peptides and proteins is also a viable conjugation strategy.

The protein C2A, containing an engineered cysteine residue, has been conjugated to THP-mal (**38**), resulting in a single equivalent of THP-mal attached per protein molecule [13]. The conjugate can be radiolabelled with ^68^Ga^3+^ at pH 5.5 (6 nmol of protein in 100 µL, at a concentration of 60 µM), giving quantitative labelling after 5 min. In vivo PET imaging in healthy mice demonstrates that ^68^Ga-THP-mal-C2A clears to the kidneys, and does not release any ^68^Ga^3+^ over a 90 min period. In contrast, in mice administered solutions of unchelated ^68^Ga^3+^, radioactivity is distributed throughout the body 90 min post-injection. In competition studies where ^68^Ga-THP-Ac is incubated with transferrin, [^68^Ga(THP-Ac)] remains intact. On the other hand, when ^68^Ga-transferrin is incubated with THP-Ac, ^68^Ga is quickly transchelated to form a complex with THP-Ac [13].

**Figure 11:**
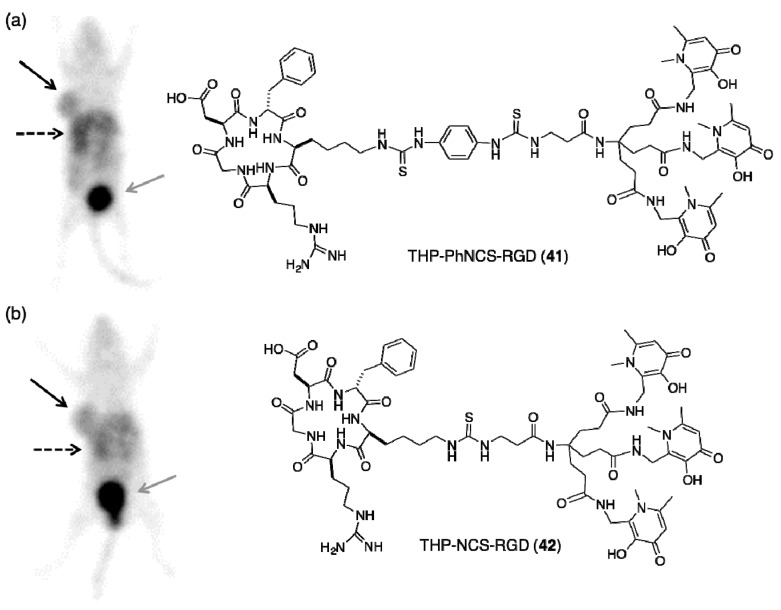
Representative PET maximum intensity projection of Balb/c nu/nu mice bearing U87MG tumours on right flank at 1 h post-injection of: (**a**) [^68^Ga(THP-Ph-NCS-RGD)]; and (**b**) [^68^Ga(THP-NCS-RGD)]. Black arrow, tumour; grey arrow, bladder; dashed arrow, kidneys and liver. Reprinted with permission under a Creative Commons Attribution (CC-BY) License from Ma, M.T.; et al. New tris(hydroxypyridinone) bifunctional chelators containing isothiocyanate groups provide a versatile platform for rapid one-step labelling and PET imaging with ^68^Ga^3+^. *Bioconjugate Chem.* **2016**, *27*, 309–318. Copyright American Chemical Society.

THP-Ph-NCS and THP-NCS have both been attached to the cyclic pentapeptide, c(RGDfK) via lysine sidechains [14]. The “RGD” peptide motif targets α_v_β_3_ integrin receptors that are expressed on the surface of many metastatic tumour cells, as well as inflamed tissue and blood vessels undergoing angiogenesis. Both THP-Ph-NCS-RGD (**41**, Figure 11) and THP-NCS-RGD (**42**, Figure 11) can be radiolabelled with generator-produced ^68^Ga^3+^ eluate at conjugate concentrations of 4–5 µM, or total amounts of 10–12 nmol, giving radiochemical yields of 95%–99%. These reactions proceed in aqueous solution, pH 5.5–6.5 in less than 5 min at ambient temperature to give a single product corresponding to either [^68^Ga(THP-Ph-NCS-RGD)] or [^68^Ga(THP-NCS-RGD)].

Both [^68^Ga(THP-Ph-NCS-RGD)] and [^68^Ga(THP-NCS-RGD)] retain affinity for α_v_β_3_ integrin receptors in vitro and in vivo [14]. PET imaging and biodistribution studies of mice bearing U87MG tumours demonstrate that both [^68^Ga(THP-Ph-NCS-RGD)] and [^68^Ga(THP-NCS-RGD)]: (i) selectively target α_v_β_3_ integrin receptors; and (ii) clear from the body within 1–2 h post-injection, predominantly via a renal route (Figure 11).

**Figure 12:**
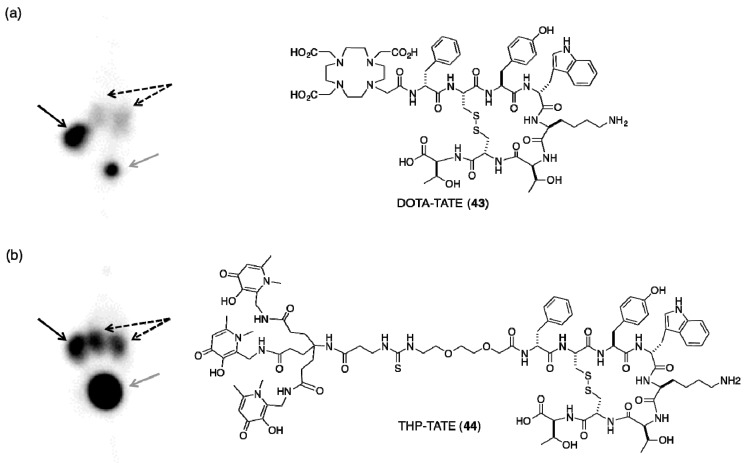
Representative PET maximum intensity projections of Balb/c nu/nu mice bearing AR47J tumours on the right flank 1 h PI of: (**a**) [^68^Ga(DOTA-TATE)]; and (**b**) [^68^Ga(THP-TATE)]. Black arrow, tumour; grey arrow, bladder; dashed arrow, kidneys. Reprinted with permission under a Creative Commons Attribution (CC-BY) License from Ma, M.T.; et al. Rapid kit-based ^68^Ga-labelling and PET imaging with THP-Tyr^3^-octreotate: a preliminary comparison with DOTA-Tyr^3^-octreotate. *EJNMMI Res.* **2015**, *5*, 52. Copyright.

The molecular imaging agent, [^68^Ga(DOTA-TATE)] is routinely used for PET imaging of neuroendocrine tumours. DOTA-TATE (**43**, Figure 12) is a conjugate of the macrocyclic chelator DOTA (**27**, Figure 9) and Tyr^3^-octreotate, an eight amino acid cyclic peptide that targets somatostatin 2 receptors (SSTR) overexpressed on the surface of neuroendocrine tumours. Using THP-NCS, THP-TATE (**44**, Figure 12) has been synthesised [15]. Similar to RGD conjugates, THP-TATE can be radiolabelled at room temperature at concentrations of 5 µM (10 nmol of conjugate) at near neutral pH in less than 2 min. In contrast, DOTA-TATE requires temperatures of 80–90 °C at pH 3–5, with reaction times of 5–10 min, and in clinical radiolabelling protocols, post-synthetic purifications procedures are invariably employed [15]. PET imaging and biodistribution experiments show that [^68^Ga(THP-TATE)] has similar uptake in SSTR-positive tumours to the clinical standard [^68^Ga(DOTA-TATE)]. [^68^Ga(THP-TATE)] clears via a renal pathway, but significantly higher kidney and liver retention of [^68^Ga(THP-TATE)] is observed (Figure 12). In PET images of mice bearing SSTR2-positive tumours administered [^68^Ga(THP-TATE)], tumours can be clearly delineated.

In all of these radiolabelled derivatives, the radiotracer demonstrates high serum stability and in vivo stability, with no evidence of dissociation of ^68^Ga^3+^ from the THP chelator.

### 5.3. Preparation, Radiolabelling and In Vitro Uptake of a Trastuzumab Immunoconjugate

New protein constructs that target receptors with high affinity and specificity have similar utility to peptides in molecular imaging. For proteins with short circulation times and rapid accumulation at target tissue, ^68^Ga PET imaging will be clinically viable, provided that appropriate radiolabelling protocols are available. The sensitivity of proteins’ tertiary structures to acidic pH and extremes of temperature requires mild radiolabelling conditions, raising problems when using conventional Ga^3+^ chelators. HBED, NOTA and DFO are capable of radiolabelling Ga^3+^ isotopes at room temperature, but HBED [63] and NOTA [64] require low pH for reactions to proceed quantitatively, and DFO complexes of Ga^3+^ are unstable [65].

Trastuzumab is a therapeutic monoclonal antibody used for treatment of breast cancer. It targets the human epidermal growth factor receptor 2 (HER2). In vivo, antibodies such as trastuzumab require extended periods of time (6 to 48 h) to clear circulation and accumulate at HER2-positive target tissue, and given the short half-life of ^68^Ga, it is likely impractical to use ^68^Ga-labelled trastuzumab to image HER2 expression. Nonetheless, it is instructive to radiolabel THP-PhNCS-trastuzumab to assess whether a THP protein conjugate that is sensitive to acidic pH (less than pH 5) can be radiolabelled rapidly under mild conditions (pH 6–7) to provide a formulation suitable for injection without further purification.

**Figure 13:**
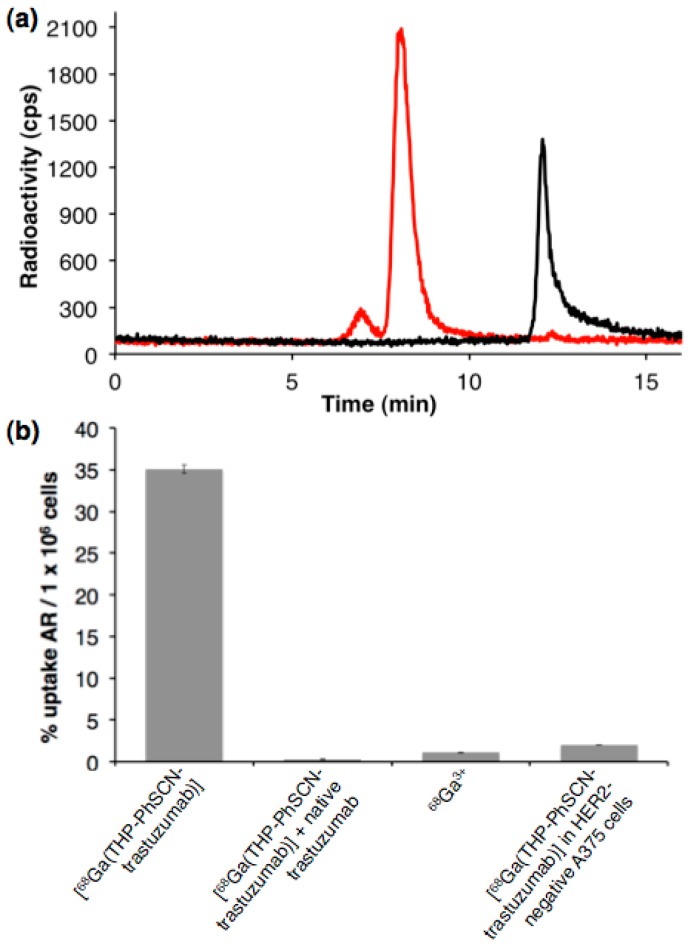
(**a**) Size exclusion radio-HPLC traces of [^68^Ga(THP-PhNCS-trastuzumab)] (**red**) and [^68^Ga(EDTA)]^−^ (**black**); and (**b**) In vitro uptake of [^68^Ga(THP-PhNCS-trastuzumab)] and [^68^Ga(THP-PhNCS-trastuzumab)] in the presence of an inhibitory concentration of trastuzumab, and ^68^Ga^3+^ in HER2-positive HCC1954 cells, and [^68^Ga(THP-PhNCS-trastuzumab)] in HER2-negative A375 cells, after 30 min incubation. Uptake is expressed as a percentage of added radioactivity (AR)/1 × 10^6^ cells (*n* = 3, error bars correspond to standard error of the mean).

The bifunctional chelator THP-PhNCS has been conjugated to the monoclonal antibody (mAb), trastuzumab by incubating a HEPES buffered solution containing both reagents under mild conditions [66,67]. The immunoconjugate, THP-PhNCS-trastuzumab has been isolated using solid phase size exclusion chromatography [66,67]. Addition of generator-produced ^68^Ga^3+^ (~10 MBq, 50 µL, aqueous 0.1 M HCl) to solutions of THP-PhNCS-trastuzumab (50 µL, 0.65 mg·mL^−1^, 0.2 M ammonium acetate, pH 6–7) results in formation of [^68^Ga(THP-PhNCS-trastuzumab)], with specific activities of up to 50 MBq·nmol^−1^ and radiochemical yields of 99% as measured by size exclusion HPLC (Figure 13a, red trace). The major signal at 7.98 min in the radiochromatogram matches the retention time of native trastuzumab, and the minor signal at 6.83 min is typical of formation of radiolabelled immunoconjugate aggregates [66]. Addition of ^68^Ga^3+^ solutions to samples containing native, unconjugated trastuzumab do not provide labelled antibody (Figure 13a, black trace)—the mobile phase in these experiments contains ethylenediaminetetraacetate (EDTA), and the single signal in the radiochromatogram with a retention time of 12.08 min corresponds to [^68^Ga(EDTA)]^−^.

To establish that the labelled immunoconjugate, [^68^Ga(THP-PhNCS-trastuzumab)], retains affinity for HER2 receptors, [^68^Ga(THP-PhNCS-trastuzumab)] has been incubated with HER2-positive HCC1954 cells and HER2-negative A375 cells. Additionally, a blockade experiment where [^68^Ga(THP-PhNCS-trastuzumab)] is incubated with HCC1954 cells in the presence of a large excess of native trastuzumab has been undertaken. Uptake of [^68^Ga(THP-PhNCS-trastuzumab)] in HCC1954 cells measures 35.12 ± 0.53 percentage of added radioactivity per one million cells (%AR/million cells), whereas uptake in A375 cells measures 1.98% ± 0.02 %AR/million cells and uptake in the trastuzumab blockade measures 0.30% ± 0.03 %AR/million, indicating that uptake of [^68^Ga(THP-PhNCS-trastuzumab)] is receptor-mediated, and that in vitro [^68^Ga(THP-PhNCS-trastuzumab)] retains affinity for HER2-expressing cells (Figure 13b). Addition of a solution containing ^68^Ga^3+^ to HCC1954 cells does not result in significant uptake of activity (1.09% ± 0.05 %AR/million cells).

Thus, THP enables efficient and rapid ^68^Ga^3+^ radiolabelling of proteins under mild, aqueous conditions. This radiolabelling strategy will have utility for ^68^Ga PET imaging of proteins such as fusion proteins and antibody fragments that have shorter clearance times than full length antibodies.

### 5.4. Other THP Derivatives

**Figure 14:**
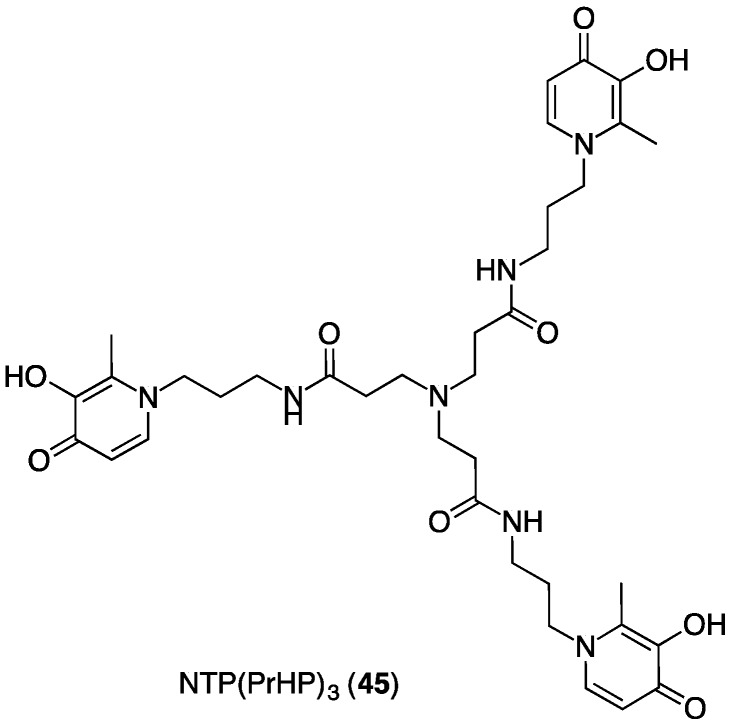
Structure of NTP(PrHP)_3_.

Alternative THP chelators such as NTP(PrHP)_3_ (**45**) have been reported for complexation of the SPECT isotope, ^67^Ga [68,69]. In these derivatives (Figure 14), the 3,4-HP groups are attached to the tripodal scaffold via the N^1^ ring atoms, and the chelating O atoms are *meta* and *para* to the linker. Such a topology can lead to formation of either dinuclear structures or structures where one 3,4-HP unit has dissociated [55]. Both species are generally more kinetically labile than species of a 1:1 stoichiometry and are not ideal for in vivo applications. The presence of more than one complex structure is also undesirable, as these species can have different biological behaviours. NTP(PrHP)_3_, with its tripodal topology centred on a tertiary amine rather than carbon, is not readily adapted to use as a bifunctional chelator. SPECT imaging and biodistribution studies with ^67^Ga^3+^ indicate that most [^67^Ga-NTP(PrHP)_3_] rapidly clears circulation within 1 h, and remaining [^67^Ga-NTP(PrHP)_3_] does not release ^67^Ga^3+^ over 24 h [69].

## 6. Hydroxypyridinones for Radiolabelling ^89^Zr^4+^

We have also investigated THP-Ac and a THP-mal-trastuzumab conjugate for radiolabelling with the long-lived PET isotope, zirconium-89 (^89^Zr^4+^) (half-life = 78 h) [16]. The Zr^4+^ ion is oxophilic, and bifunctional hexadentate DFO derivatives are most commonly utilised for incorporating ^89^Zr^4+^ into antibodies. There is evidence that [^89^Zr(DFO)]^+^ derivatives are unstable in vivo, with observations of ^89^Zr accumulation in the skeleton over the course of a week [16]. Here, skeletal uptake is assumed to be indicative of dissociation of ^89^Zr^4+^ from a chelator-conjugate.

**Figure 15:**
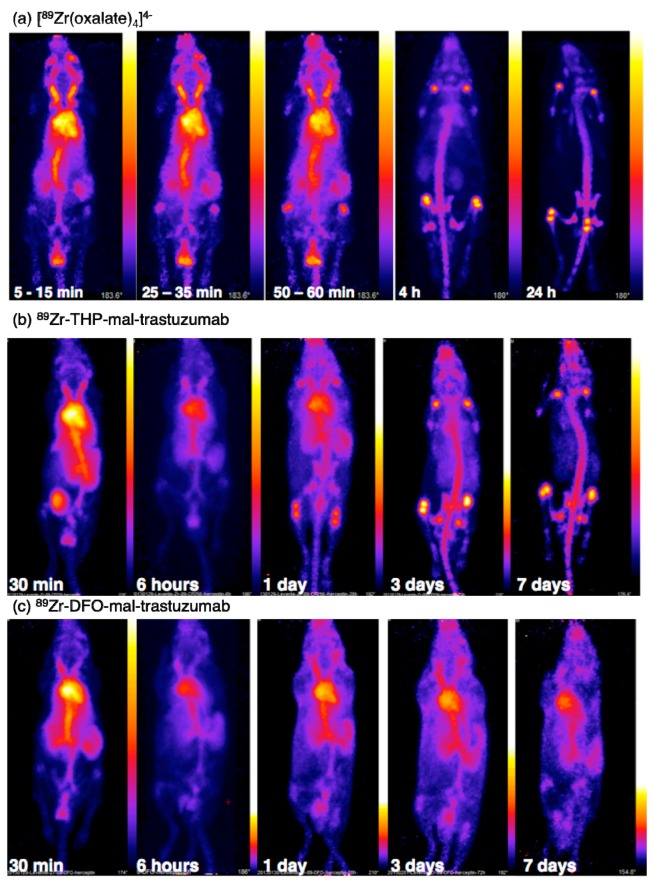
PET scans of C57Bl/6j mice administered: (**a**) [^89^Zr(oxalate)_4_]^4−^; (**b**) ^89^Zr-THP-mal-trastuzumab; and (**c**) ^89^Zr-DFO-mal-trastuzumab. For the animal administered [^89^Zr(ox)_4_]^4−^ 98% of the injected dose remains 4 h post-injection, and is associated predominantly with the skeleton. For the animal administered ^89^Zr-THP-mal-trastuzumab, the skeleton is clearly visible from three days post-injection, indicative of dissociation of ^89^Zr^4+^ from THP-mal-trastuzumab over time. This is in marked contrast to the animal administered ^89^Zr-DFO-trastuzumab, where the blood pool remains visible out to seven days post-injection. Reprinted with permission under a Creative Commons Attribution (CC-BY) License from Ma, M.T.; et al. Tripodal tris(hydroxypyridinone) ligands for immunoconjugate PET imaging with ^89^Zr^4+^: comparison with desferrioxamine-B. *Dalton Trans.* **2015**, *44*, 4884–4900. Copyright Royal Society of Chemistry.

Both THP-Ac and THP-mal-trastuzumab can be radiolabelled with ^89^Zr^4+^ at room temperature and near neutral pH in quantitative yield. For THP-Ac, this was achieved at 1 mM concentration and 10 µL volume, corresponding to 10 nmol of ligand. Competition experiments indicated a thermodynamic preference for Zr^4+^ coordination to THP-Ac over DFO. This is consistent with higher metal ion affinities of 3,4-HPs compared to hydroxamates. However, experiments in which ^89^Zr-THP-mal-trastuzumab was administered to healthy animals indicate that in vivo, ^89^Zr^4+^ dissociates from THP-mal-trastuzumab within 48 h, with activity accumulating in the skeleton (Figure 15). In vivo stability of the [^89^Zr(THP)]^+^ complex is inferior to that of the [^89^Zr(DFO)]^+^ complex, possibly a result of the different chelator topologies.

Zr^4+^ can accommodate up to eight donor atoms in its coordination sphere, and these coordination requirements are likely to be a factor in the instability of [^89^Zr(THP)]^+^ in vivo. The presence of two coordination sites unoccupied by THP chelator allows coordination of endogenous ligands, and provides pathways to transmetallation/ligand exchange.

**Figure 16:**
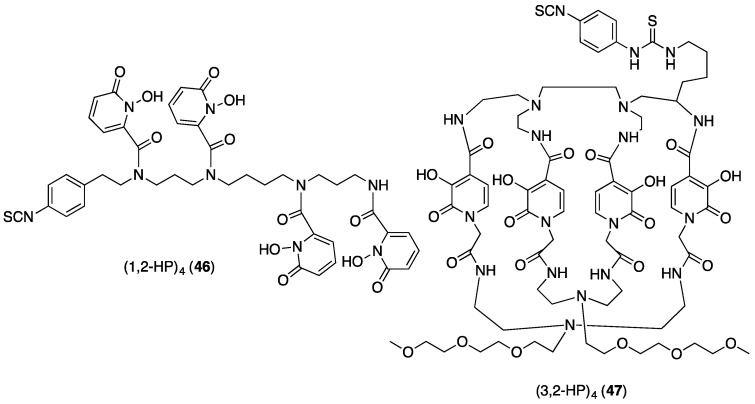
Structures of octadentate (HP)_4_ derivatives evaluated for ^89^Zr^4+^ immunoconjugate radiolabelling.

It is possible that a tetrakis(3,4-hydroxypyridinone) ligand could impart greater in vivo stability, but there are no reports of such ligands to date. Other researchers have studied octadentate tetrakis(hydroxypyridinone) derivatives for ^89^Zr^4+^ incorporating 1,2-HPs and 3,2-HPs. The bifunctional open chain (1,2-HP)_4_ chelator (**46**, Figure 16) can: (i) coordinate Zr^4+^ in an octadentate environment; and (ii) be conjugated to trastuzumab. Immunoconjugate (1,2-HP)_4_-trastuzumab can be radiolabelled with ^89^Zr^4+^ at room temperature, and PET imaging and biodistribution studies demonstrate high in vivo stability of the complex, with concomitant high tumour uptake [70].

Bifunctional macrobicyclic octadentate (3,2-HP)_4_ (**47**, Figure 16) has also been derivatised for conjugation to trastuzumab [71]. The coordination environment of the Zr^4+^ complex is not well defined, and chromatographic analysis indicates that multiple Zr-bound (3,2-HP)_4_ species form under the mild reaction conditions described (room temperature, 15 min incubation). PET imaging and biodistribution studies in mice using ^89^Zr-labelled (3,2-HP)_4_-antibody conjugates suggest that at least one form of ^89^Zr^4+^-(3,2-HP)_4_ is unstable in vivo, as significantly higher bone uptake is observed for (3,2-HP)_4_ conjugates compared to DFO conjugates. This highlights the difficulty in interpreting in vivo results based on radiolabelled compounds that contain metal complexes in more than one conformation.

## 7. Concluding Remarks

Derivatisation of 3,4-HPs via substitution of ring protons allows for tailoring of chelator properties including the proton and Fe^3+^ affinities, and in vivo distribution and reactivity towards endogenous enzymes. 3,4-HPs can be further functionalised with fluorescent tags and biologically active motifs, and can be incorporated into chelators of higher denticity. Hexadentate THP chelators based on 3,4-HPs possess extraordinarily high pFe^3+^ values, and are very potent Fe^3+^ scavengers. As Fe^3+^ and Ga^3+^ have similar coordination preferences, THP chelators are ideal candidates for development of PET radiopharmaceuticals based on ^68^Ga^3+^, and direct translation of this chemistry has enabled rapid development of bioconjugates of THP chelators for ^68^Ga PET imaging.

Simplicity of radiolabelling with minimal need for complex equipment and radiochemical expertise is likely to be a key to the wider availability of ^68^Ga PET, and this is afforded by appropriate design of a ^68^Ga chelator. THP derivatives fulfil these requirements whereas other established chelator designs do not. Current clinical radiosynthetic protocols based on DOTA and HBED derivatives require heating (>80 °C), low pH (3–5) and post-synthetic purification/formulation. In contrast, THP compounds can be radiolabelled and formulated by treatment with generator-produced ^68^Ga^3+^ in over 95% radiochemical yield under ambient conditions in less than 5 min, at low chelator concentrations, neutral pH and in aqueous solution. There is no requirement for post-synthetic purification or reformulation, as reactions are quantitative and components (solvents and buffers) are physiologically compatible. Bifunctional THP derivatives enable a means of attachment to peptides and proteins, and biological studies have demonstrated that the peptide radiotracers are stable in vivo with respect to dissociation of the ^68^Ga-THP complex, retain affinity for target receptors and clear rapidly from circulation. All of these characteristics make THP superlative for one-step kit-based radiosynthesis.

## Figures and Tables

**Table 1:** Protonation constants, stepwise stability constants (log *K*) and cumulative Fe^3+^ stability constants (log *β*_3_) for 1,2-HP, 3,2-HP and 3,4-HP.

Compound	p*K*_a1_	p*K*_a2_	log *K*_1_	log *K*_2_	log *K*_3_	log *β*_3_ Fe^3+^	Ref.
1,2-HP (**2**)	1.2	5.86	10.6	9.5	7.1	27.2	[22]
	−0.9	5.78	10.3	9.0	7.6	26.9	[23]
3,2-HP (**3**)	0.1	8.66	11.7	9.8	8.1	29.6	[23]
3,4-HP (**4**)	3.34	9.01	14.2	11.6	9.3	35.1	[23]
	3.60	9.60	–	–	–	36.9	[20]

**Table 2:** Protonation constants and Fe^3+^ and Ga^3+^ stability constants for deferiprone (**1**) (measured at 22.5–25 °C, ionic strength 0.1–0.2 M; for pM values, pH 7.4, with [total ligand] = 10^−5^ M and [total metal ion] = 10^−6^ M).

Metal Ion	p*K*_a1_	p*K*_a2_	log *K*_1_	log *K*_2_	log *K*_3_	log *β*_3_	pM^3+^	Ref.
Fe^3+^	3.56	9.64	14.92	12.23	9.79	37.2	–	[20]
	3.68	9.77	14.56	12.19	9.69	36.4	19.4	[34]
	3.62	9.76	15.14	11.54	9.24	35.92	18.3	[35]
	–	–	15.10	11.51	9.27	35.88	–	[25]
	3.61	9.78	15.03	27.42	–	37.35	20.74	[36]
Ga^3+^	–	–	13.17	12.26	10.33	35.76	–	[25]
	3.70	9.86	17.07	12.19	9.16	38.42	–	[37]

**Table 3:** Protonation constants and Fe^3+^ stability constants for 3,4-HP derivatives.

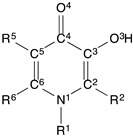									
Compound	R^1^	R^2^	R^5^	R^6^	p*K*_a1_	p*K*_a2_	log *β_3_* (Fe^3+^)	pFe^3+^	Ref.
**1**	CH_3_	CH_3_	H	H	3.68	9.77	36.4	19.4	[34]
					3.61	9.78	37.35	20.74	[36]
**4**	H	H	H	H	3.34	9.01	35.1	–	[20,23]
**5**	H	CH_3_	H	H	3.70	9.76	37.2	–	[20]
					3.64	9.73	36.63	20.17	[36]
**6**	CH_3_	CH_2_OH	H	H	2.92	9.11	35.3	20.9	[34]
**7**	CH_2_CH_3_	CH_2_CH_3_	H	H	3.81	9.93	36.8	19.7	[34]
**7**	CH_2_CH_3_	CH_2_OH	H	H	2.80	9.27	35.3	21.0	[34]
**9**	CH_2_CH_3_	CH(OH)CH_3_	H	H	3.03	8.77	35.1	21.4	[34]
**10**	H	CON(CH_3_)_2_	H	CH_3_	2.53	8.20	33.2	20.4	[38]
**11**	H	CONHCH_3_	H	CH_3_	6.66	2.32	32.5	22.8	[38]
**12**	CH_2_CH_3_	CH_3_	H	H	3.65	9.88	37.7	–	[20]
**13**	CH_3_	CH_3_	CH_3_	H	3.37	10.32	37.93	19.71	[36]
**14**	H	CH_3_	CH_3_	H	3.43	10.27	37.28	19.22	[36]

## Abbreviations

Bn	Benzyl
DFO	Deferrioxamine B
EDTA	Ethylenediaminetetraacetate
GIT	Gastrointestinal tract
HER2	Human epidermal growth factor receptor 2
HP	Hydroxypyridinone
HPLC	High performance liquid chromatography
PET	Positron Emission Tomography
RGD	Cyclic RGDfK peptide
TATE	Octreotate
THP	Tris(hydroxypyridinone)
SSTR2	Somatostatin 2 receptor
